# Prediction Model of the Resulting Dimensions of Welded Stamped Parts

**DOI:** 10.3390/ma14113062

**Published:** 2021-06-03

**Authors:** Milan Kadnár, Peter Káčer, Marta Harničárová, Jan Valíček, Mirek Gombár, Milena Kušnerová, František Tóth, Marian Boržan, Juraj Rusnák

**Affiliations:** 1 Department of Machine Design, Faculty of Engineering, Slovak University of Agriculture in Nitra, Tr. A. Hlinku 2, 949 76 Nitra, Slovakia; milan.kadnar@uniag.sk (M.K.); kacer.pet@gmail.com (P.K.); frantisek.toth@uniag.sk (F.T.); juraj.rusnak@uniag.sk (J.R.); 2 Department of Electrical Engineering, Automation and Informatics, Faculty of Engineering, Slovak University of Agriculture in Nitra, Tr. A. Hlinku 2, 949 76 Nitra, Slovakia; jan.valicek@uniag.sk; 3 Department of Mechanical Engineering, Faculty of Technology, Institute of Technology and Business in České Budějovice, Okružní 10, 370 01 České Budějovice, Czech Republic; kusnerova.milena@mail.vstecb.cz; 4 Faculty of Management, University of Prešov in Prešov, Konštantínova 16, 080 01 Prešov, Slovakia; miroslav.gombar@unipo.sk; 5 Department of Manufacturing Engineering, Faculty of Machine Building, Technical University of Cluj-Napoca, B-dul Muncii, No. 103-105, 400641 Cluj-Napoca, Romania; mborzan@yahoo.com

**Keywords:** welding, distortion, stamping, model, prediction

## Abstract

The combination of stamping and subsequent welding of components is an important area of the automotive industry. Stamping inaccuracies affect the final size of the stamping and the welded part. In this article, we deal with a specific component that is produced by such a procedure and is also a common part of the geometry of a car. We focused on the possibility of using a negative phenomenon—deformation during welding—on the partial elimination of inaccuracies arising during stamping. Based on the planned experiment, we created a prediction model for the selected part and its production, with the help of which it is possible to determine suitable welding parameters for a specific dimension of the stamping and the required monitored dimension of the welded part. The article also includes the results of additional experimental measurements verifying the accuracy of the model and prediction maps for practice.

## 1. Introduction

Welding is the most important method of joining metal components. There are many joining techniques available. Each technology is specific and has its advantages or disadvantages for a certain application. The sectors where welding plays a dominant role include the automotive industry. The most common welding techniques in the automotive industry are laser beam welding [1], metal inert gas welding process, metal active gas welding process [2], and spot welding [3].

MIG (metal inert gas) welding process was used in this study. MIG welding is a remarkably flexible method. The principle of MIG welding is that the melted bath is protected from the effects of the surrounding atmosphere (mainly oxygen and nitrogen) by a protective atmosphere, which may be inert or active. Inert atmospheres do not enter into chemical reactions with the melting bath. Active atmospheres participate in chemical reactions in the melting bath; their action is being compensated by a suitable composition of the additive material [4,5]. Weld heat input is important, because it affects the amount of distortion and residual stress in the component. The problems of these thermal effects significantly affect manufacturers in the automotive industry, because the welded assembly of the car must be kept in tight tolerances [6].

**Figure 1:**
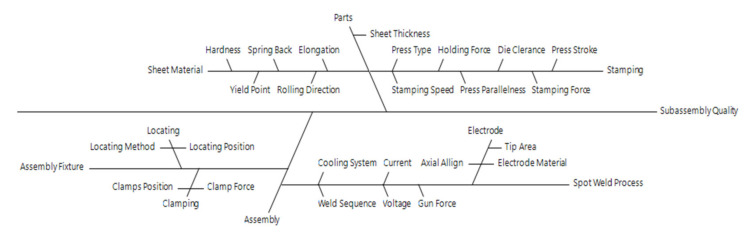
Ishikawa diagram for the identification of the subassembly quality of a car [9].

The vehicle body is created by several stamped components, which are joined together by the spot-welding process. The quality of the joint is determined by its geometry as well as by its local properties (mechanical, microstructural, chemical, etc.). Problems with general weldability can be solved with the right choice of materials suitable for welding, the design of welding technology suitable for the selected material, and the implementation of appropriate design modifications needed for successful welding. It follows that weldability is influenced by three main groups of interrelated factors, material–design–technology. The interconnection of the three main factors cannot be divided and assessed independently but must always be assessed comprehensively. In manufacturing a car, a combination of different technological processes often takes place, whereas a considerable number of factors influence the resulting parameters of the manufactured component. The typical combination of pressing and subsequent welding of stamped parts is an area that still deserves increased attention. Cost optimisation and efficient use of technological equipment in the case of small and medium-sized enterprises lead to an operational change in production. In the case of pressing, this means that a tool is often changed to ensure the production of different products. When changing tools, height adjustment deviations can occur due to imperfectly clean loading surfaces and mounting clearances. These directly affect the height of the press and, thus, the size of the stamped part [7]. Steel coils, where the changes in the chemical composition occur, also have a significant effect on the dimensions. Therefore, in the subsequent welding operation, the size of the stamped part represents the input factor [8]. The major causes of the dimensional inconsistencies in vehicle body components can be classified in the following points: assembly operations and positional capabilities, material properties (including the history of material production), stamping process parameters, and the welding process, itself (Figure 1) [9].

**Figure 2:**
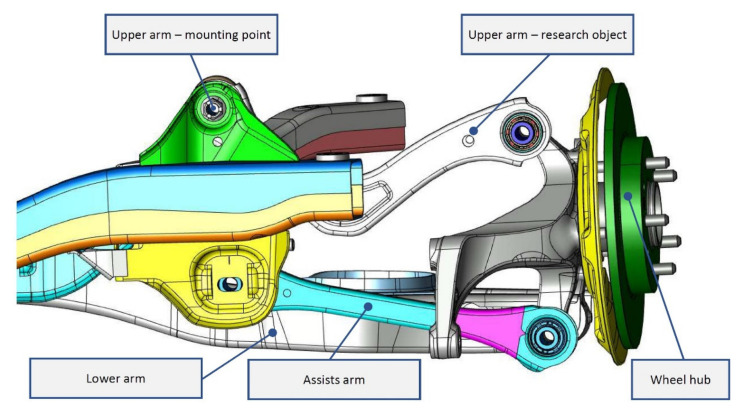
Alignment corrections.

Over the last several years, there has been a rising demand for quality in the automotive industry, which forces manufacturers to solve considerable problems [10]. One of them is the assembly and setting of wheel geometry parameters. When the prescribed dimensional values are not observed, it can lead to problems that are reflected in the difficult or impossible deflection/tilt setting. Negative/positive tilt/deflection negatively affect the driving characteristics of the car. The car is not suitable for sale, but it is intended for repair. The dimensional deviations of certain parts and their joints affect the geometry the most. In this paper, we deal with the use of the negative phenomenon of deformation during welding to eliminate the differences caused by pressing a particular component, which is part of the geometry of a car (Figure 2). 

Several studies have been made to study the effects of welding process parameters on the resulting geometry. Hamedi et al. [9] studied the effects of spot-welding parameters (current, time, and gun force) on the deformation of the subassemblies and the overall quality of the car body. They used a neural network and multi-objective genetic algorithm to find out the optimum values in order to get the least values of dimensional deviations in the subassemblies. Similar work was done by Kim et al. [11], who dealt with the response surface methodology to optimise the welding current, welding time, and welding force as input parameters and shear strength and indentation as output parameters. The finite element method (FEM) has been used for this purpose by Caro et al. [12]. The sheet metal forming procedure of a double-curved component made of alloy 718 has been studied using the FE method. This approach seems to be suitable for predicting distortions located in the same places as found during the experiment. Thomas et al. [7] confirmed the presumption of suitability for the use of the finite element method for accurate predictions of the final shape of stamped automotive assemblies, including the springback deformation of parts. Li et al. [13] studied the relationship between the weld quality and various process conditions using a two-stage, sliding-level experiment. A detailed description of the statistical analysis is shown in Zhang et al. [14] for predictions of expulsion limits. Muthu [4] performed an analysis of variance (ANOVA) to find the most significant parameters affecting the spot weld quality characteristics. An alternative approach based on the Taguchi method was used to analyse every welding process parameter for obtaining optimal weld pool geometry [15]. Other researchers [16,17] have attempted to find the optimal welding parameters by artificial neural networks (ANN).

Welding process failures and dimensional changes are the main leading factors to a decrease in productivity. Deformation induced by welding has many negative effects, one of which affects the dimension accuracy. The aim is to minimise the welding deformation. To mitigate this problem, the predictions play an important role to improve manufacturing accuracy. Statistical process control is often used to control the process in order to keep a high dimensional quality of the product [18].

Most published papers are focused on using the finite element method (FEM) to perform engineering analysis. FEA models have an advantage: they consider the effects during the welding, such as the phase transformations and the transformation strains during cooling. Some procedures of a dimensional control in the full automotive body are shown in [19,20,21]. The progress has been made also by developing the structure analysis method [22], the knowledge-based and model-based diagnostics techniques or tolerance analysis based on a mechanistic model [23].

Based on the literature search, it can be stated that the use of deformation during welding to eliminate inaccuracies caused by pressing has not been addressed so far. Therefore, in the paper, we present the original results of welded part deformation measurements (Donghee Slovakia, Ltd., Strečno, Slovakia), whose complex shape and changing dimensions limit the possibility of determining the optimal welding parameters. The influence of the basic process parameters, namely the welding current and the welding speed in combination with the changing size of the stamping, was verified. The presented original methodology and prediction model in practice allow welders to control the final dimension of the welded part with great accuracy and to respond to dimensional or capacity requirements of the production.

## 2. Materials and Methods

**Figure 3:**
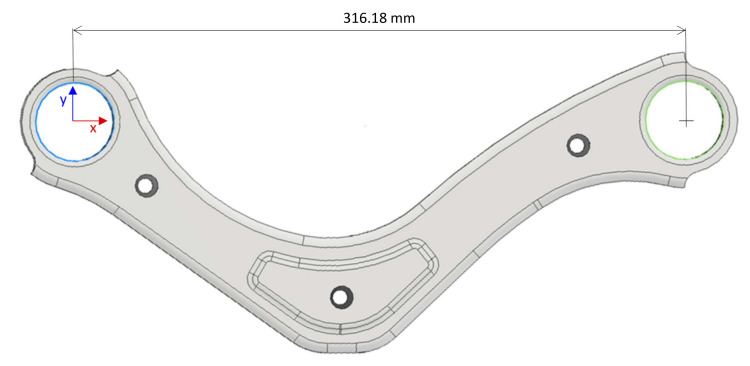
A welded part and the dimension to be examined.

The object of research is a welded part (2 stampings)—see an example in Figure 3. Its thickness is 24 mm, and the dimension to be checked is supposed to be 315.5 ± 0.2 mm. In this particular case, the whole series of stampings is 0.68 mm larger than the nominal value.

**Figure 4:**
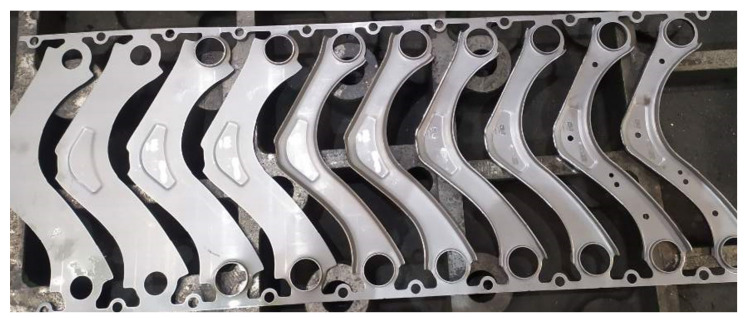
Stamping procedure.

The stampings are stamped on a SIMPAC MC2-500 press using a progressive 13-operation mould (Figure 4). The size of the stamping is affected by the setting of the press and the tool, and, during the series, the size is constant and regularly checked. After switching to a new series, the size of the stamping changes directly affects the resulting size of the welded part.

All examined samples were stamped from Dual phase-type steel SGAFC590DP. Chemical properties are given in Table 1 and mechanical properties in Table 2. The values were obtained from the material sheet from the company Hyundai Steel Co., Ltd. (Seoul, Korea).

As with any process, the welding process is significantly affected by the process setting parameters. There are a number of associated parameters with this technology, among which the current, voltage, and welding speed are significant and precisely controllable. The OTC DM-400 welding machine (OTC Daihen Europe, GmbH., Mönchengladbach, Germany) deployed in combination with the ALmega AX V6 welding robot (OTC Daihen Europe, GmbH., Mönchengladbach, Germany) offers the possibility of automatic voltage determination. Since this is used in practice, we decided to consider this parameter constant (due to the multicollinearity of the model). In the experiment, we considered both current and welding speed to be the variables. A summary of welding parameters is given in Table 3. 

**Figure 5:**
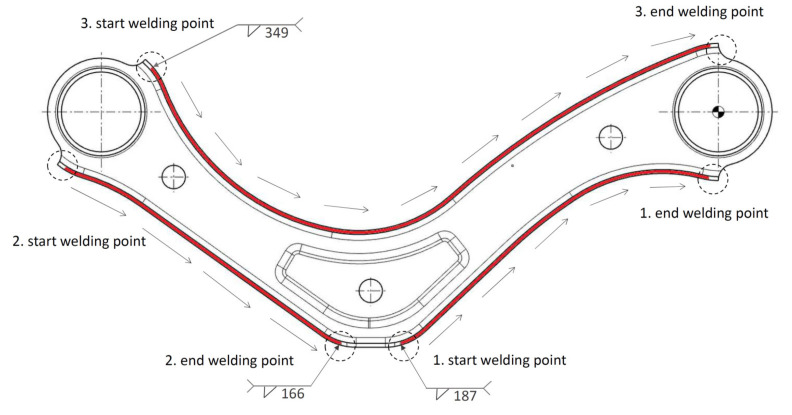
Location of welds and their order during welding.

**Figure 6:**
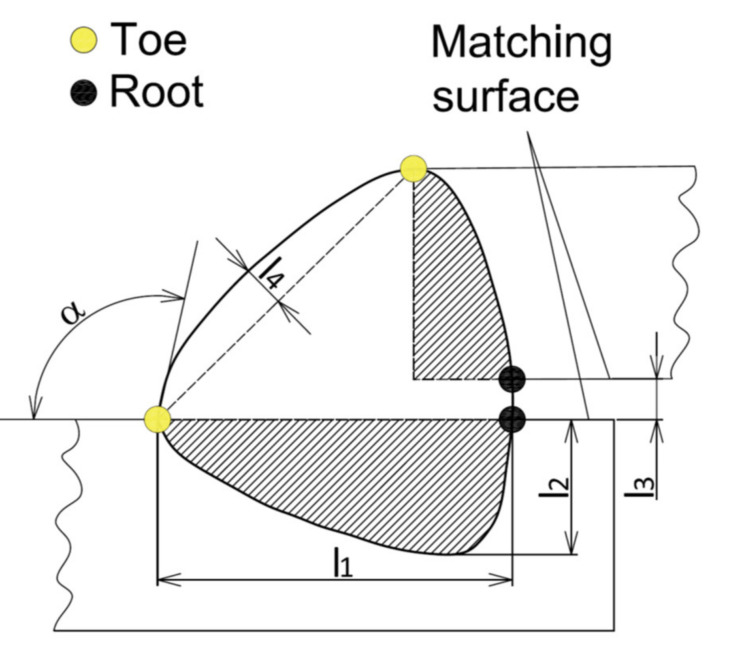
Geometrical shape of weld joint with the monitored dimensions in quality inspection (α—toe angle; l_1_—leg length; l_2_—penetration; l_3_—gap width; l_4_—excess welding).

The location of the welds and their order during welding are shown in Figure 5 and the geometrical shape of lap weld joint in Figure 6.

The parameters used during the measurements were given by a combination of welding speed from 50–70 cm·min^−1^ and electric current 160–200 A, while each change in current also resulted in a change in the corresponding voltage (automatically). The currents 180–200 A are the most used in this production setting due to the lower error rate and, at the same time, due to the possible higher welding speeds.

**Figure 7:**
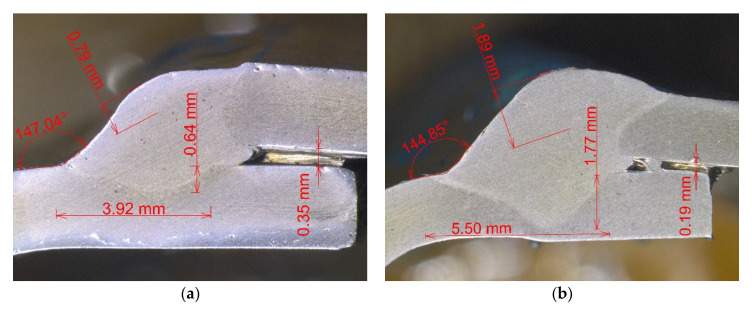
Weld cut: (**a**) Welding parameters *I* = 160 A, *v* = 70 cm·min^−1^; (**b**) welding parameters *I* = 200A, *v* = 50 cm·min^−1^.

Lower current values, i.e., 160–180 A proved to be equally applicable; the values lower than 150 A required a lower welding speed due to the correct weld of the material, which is not applicable in technical practice. From the point of view of the experiment, it was also important that the deformations were minimal to zero at the level lower than 150 A in the preliminary tests. Sections of welds realized under the boundary conditions of the experiment from the point of view of the introduced heat are shown in Figure 7.

**Figure 8:**
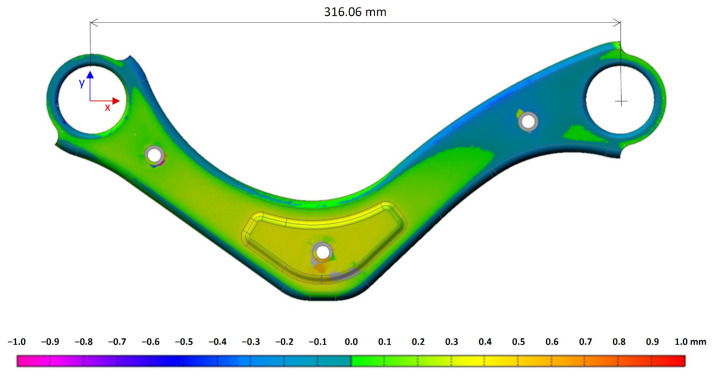
The total deviation of the stamped component from the 3D model.

**Figure 9:**
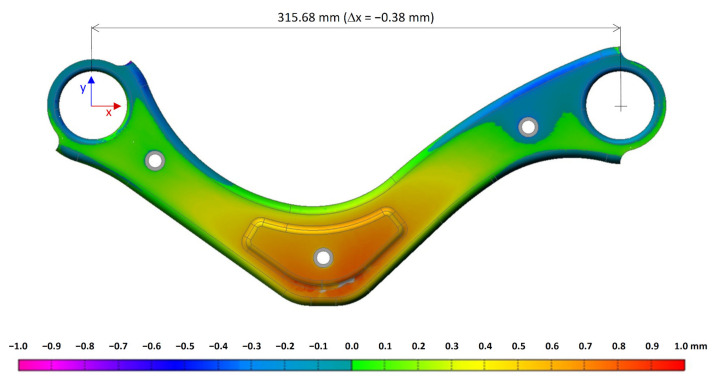
The total deviation of the welded part from the 3D model. Welding parameters (*I* = 190 A, *v* = 60 cm·min^−1^).

The total deviation of the component (against 3D model, measured on a Romer Absolute Arm device) before and after welding are shown in Figure 8 and Figure 9. The colour map represents the spatial deformation, and the dimension is monitored.

**Figure 10:**
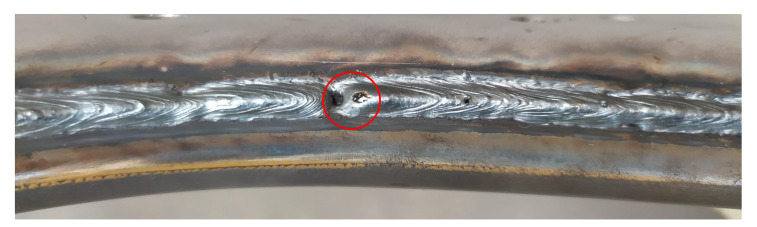
Defects when using incorrect welding parameters (*I* = 210 A, *v* = 80 cm·min^−1^).

Currents of a bigger magnitude than 200 A cause a high error rate (burn-trough) since, at high currents, a high speed must be selected, which also places great demands on accuracy. This cannot be achieved with the current technologies used. When tested at these levels, the results were very unstable, and the welds were of poor quality (holes in the welds, Figure 10), which had a negative effect on the overheating of the material and also on the deformation.

In order to determine the influence of selected factors on the final dimension of the welded part, an experiment was performed, and statistical analysis of the experimentally obtained data was performed. The influence of three technological factors such as welding current, welding speed, and stamping size were examined. The experiment was carried out according to a partial central composite design, and, due to the significant effect of axial points on the resulting weld quality, the Face Centered variant of axial points was chosen. Pseudo-central points were also part of the plan, as it is not possible to provide measurements at the central level for factor *x*_3_ for operational reasons. Levels of factor *x*_3_ were selected based on long-term observations at the lower and upper limit of the produced stampings. The levels of factors *x*_1_ and *x*_2_ were chosen with respect to the penetration tests of welds performed during long-term observations, the standard values of these factors, and the desired effect—reducing the size of the welded part. The selected factor levels are given in Table 4 and the standard levels in Table 5. In addition to these factors, the experiment was performed under the same welding conditions, and all parts that entered welding were from one stamping batch (for a specific value *x*_3_).

**Figure 11:**
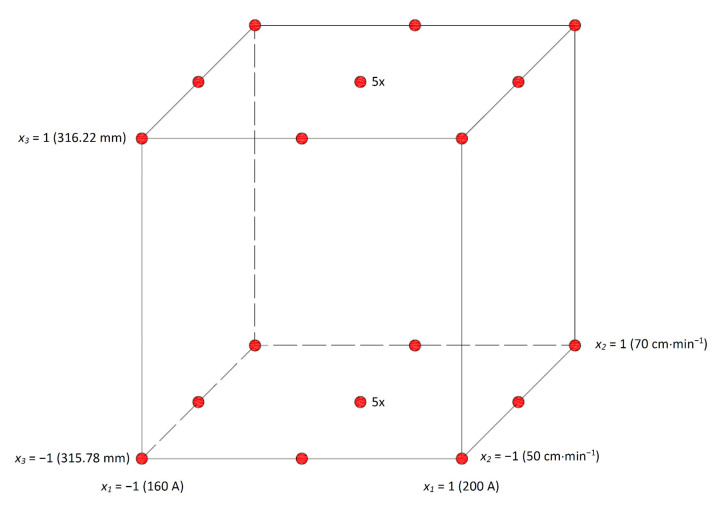
Demonstration of the proposed experiment plan.

The experimental design included a total of 8 cube points, 8 axial points, and 10 pseudo-central points. A graphical representation of the experimental design (with five replicates at pseudo-central points) is shown in Figure 11.

In addition to the plan, measurements were performed in order to assess the accuracy of the model for the parameters listed in Table 6.

## 3. Results and Discussion

When analysing the individual levels of factors and their influence on the resulting length of the welded arm using nonparametric Kruskal–Wallis analysis [24,25] of variance (Table 7), it can be stated that

- at the significance level of 5%, the welding current ((H 2, N = 99) = 6.5254) is a significant factor influencing the change in arm length (*p* = 0.0383),
- at a significance level of 5%, the welding speed ((H 10, N = 99) = 19.7374) is a significant factor influencing the change in arm length (*p* = 0.0318), and
- at the level of significance of 5%, the size of the stamping ((H 2, N = 99) = 70.1071) is a significant factor influencing the change in the length of the arm (*p* < 0.001).

Further analysis by multiple comparisons of *p* values shows that there is a statistically significant difference in the value of the welded arm length at a current value of 160 A and a value of 200 A. Increasing the current value by 40 A causes a decrease in the arm length value by 0.14 mm. The difference between the value of the length of the welded arm at a current of 180 A and 200 A represents a value of 0.07 mm, but this difference is not significant at the significance level of 5%.

Statistically significant differences in the arm length at different speed values are given in Table 8.

**Figure 12:**
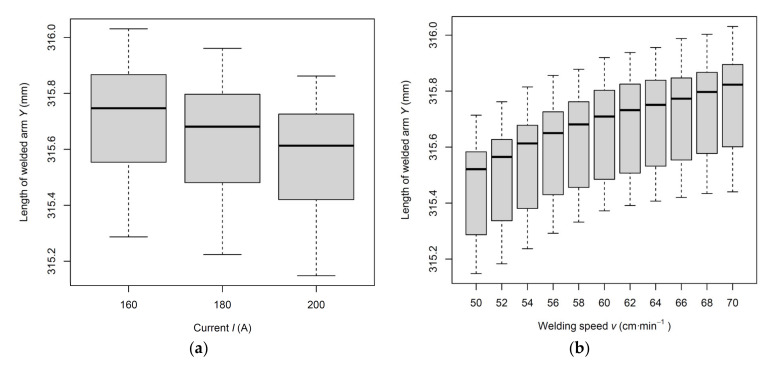
The influence of factor levels on the mean response Y value: (**a**) current *I* (A); (**b**) welding speed *v* (cm·min^−1^).

Based on the analysis performed by multiple comparisons of *p* values, it can be further stated that a statistically significant difference was seen in the value of the length of the welded arm at the size of the stamping 315.78 mm and the value 316.22 mm. Increasing the value by 0.44 mm increased the value of the arm length by 0.43 mm. The difference between the value of the length of the welded arm for the 316.08 mm and 316.22 mm stamping is 0.14 mm, and this difference is also significant at the significance level of 5%. The influence of the *I* and *v* parameter levels on the mean value of the response *Y* is shown in Figure 12.

The degree of overlap, e.g., between the values at the minimum and maximum current level given by the degree of influence of the given factor on the mean value of the response. The overall variability of the response is given by the number of factors and the selection of their levels, and the more significant the influence of the factor on the mean value of the response, the smaller the overlap. From the point of view of the model, this influence is illustrated by the estimation of the model parameters. The smaller the estimated value (influence) of a given factor, the greater the overlap between levels.

Statistical modelling by regression analysis was applied to create a complex dependence of the experimentally investigated welding factors on the value of the final length of the welded component *Y*. The basic results for the required dependence in a general form can be expressed by Equation (1).

(1) $$Y=f\left(x_{1},x_{2},x_{3}\right)$$

where *x*_1_—electric current [A], *x*_2_—welding speed [cm·min^−1^], and *x*_3_—stamping dimension [mm]. The suitability of the used model is documented in Table 9.

The results show that the predictive power of the model expressed by the adjusted index of determination represents a value of 96.9%. Thus, the model cannot explain 3.1% of the variability of the investigated parameter of the length of the welded arm *Y*. The average value of the length of the weldment represents a value of 315.612 mm with an average error of 0.044 mm.

From the table of the variance analysis (Table 10), it can be concluded that the variability caused by random errors is significantly less than the variability of the measured values explained by the model. Model F Ratio value implies the model is significant. There is only a 0.01% probability that such a significant F value could occur due to noise.

Testing the null (H_0_) statistical hypothesis [26,27], which results from the nature of the Fisher–Snedecor test criterion, allows us to conclude that, based on the achieved level of significance *p* = 0.0001, the null hypothesis of the Fisher–Snedecor test criterion can be rejected. It is possible to accept the alternative hypothesis that there is at least one factor whose regression coefficient is statistically different from zero and thus significantly affects the change of the investigated parameter *Y*. This means that adequate input variables were chosen to describe the change in the dimension of the welded part *Y*. The model in terms of Fisher–Snedecor test criterion is adequate and significant.

Further testing of the model used was carried out by the so-called lack of fit error test, i.e., the variance of residues and the variance of the measured data within the groups were tested. Thus, it is tested whether the regression model sufficiently captures the observed dependence (Table 11). 

Given the significance value of 1000 achieved by the Fisher test, a null statistical hypothesis can be accepted for the observed variable *Y*, which results from the nature of the mismatch error test, and we can say that the model sufficiently captures the variability of experimentally obtained data at the 5% significance level.

Based on the above assumptions and their fulfilment (Table 10 and Table 11), the following table (Table 12) presents an estimate of the model parameters with testing the significance of individual effects and their combination at the significance level *α* = 0.05.

The table for estimating the parameters of the model (Table 12) shows that the welding current on the significance level of 5% has a significant effect on the change in the values of the investigated parameter *Y* in the range of experimentally used input variables and influence of this input investigated variable on the total value *Y* variability. Furthermore, it can be concluded that, as the current increases, the conditional arm length value *Y* also decreases. Another significant input parameter that affects the arm length value is the welding speed, with an overall effect on the variability of the *Y* value of 32.35%. The influence of the stamping size is also significant, with 55.59% influence on the change in the arm length value *Y*. It follows from the above that, in the monitored parameter range, the stamping dimension has the most significant influence. In contrast, with other parameters, its influence can be corrected significantly, but not completely.

The output of the model is coded Equation (2)

(2) $$Y=315.634-0.069\cdot x_{1}+0.151\cdot x_{2}+0.217\cdot x_{3}-0.0485119\cdot x_{2}^{2}$$

**Figure 13:**
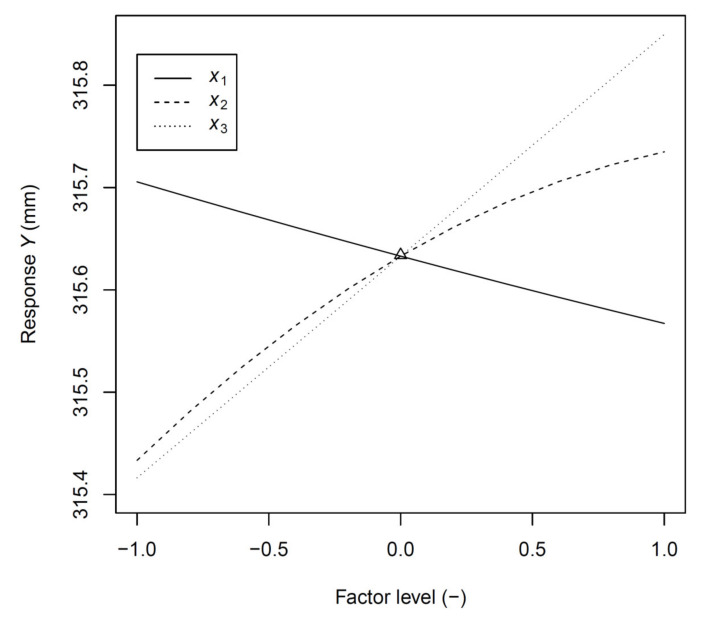
Influence of individual factors on the resulting Response *Y*.

The first equation term (intercept) represents the centre of design space. It is clear from the equation that there is a positive correlation between the dimension of the stamping *x*_3_ and the resulting value of the length of the arm *Y*. Conversely, there is a negative correlation between the welding current *x*_1_ and *Y*. Thus, the increasing current causes a more significant deformation of the arm, and thus, the resulting arm length *Y* decreases. The influence of the welding speed *x*_2_ on the resulting value is determined by two members of the equation, while the linear term has a positive correlation and the quadratic term negative correlation. Due to the size of the coefficients of the given members of the equation, the resulting correlation between *x*_2_ and *Y* is positive. Thus, with increasing welding speed, the total length of the component *Y* also increases, i.e., the deformation during welding is smaller in comparison. A graphical representation of the resulting influence of individual factors on the value *Y* is shown in Figure 13.

Based on the above facts, a regression dependence can finally be predicted (3):

(3) $$Y=2.503-3.458\cdot 10^{-3}\cdot I+7.209\cdot 10^{-2}\cdot v+9.846\cdot 10^{-1}\cdot Z-4.750\cdot 10^{-4}\cdot v^{2}$$

This equation is suitable for determining predictions on a scale of selected intervals for individual factors, i.e., for technical practice. At the same time, however, because the intercept is not the centre of design space and the regression coefficients are modified by conversion from coded to actual scale, the equation is not suitable for determining the influence of individual factors for interpreting the model.

**Figure 14:**
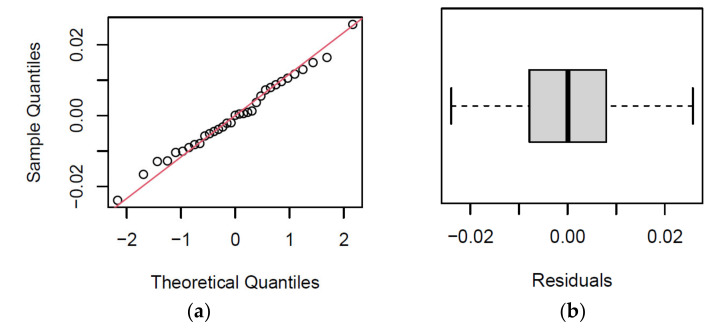

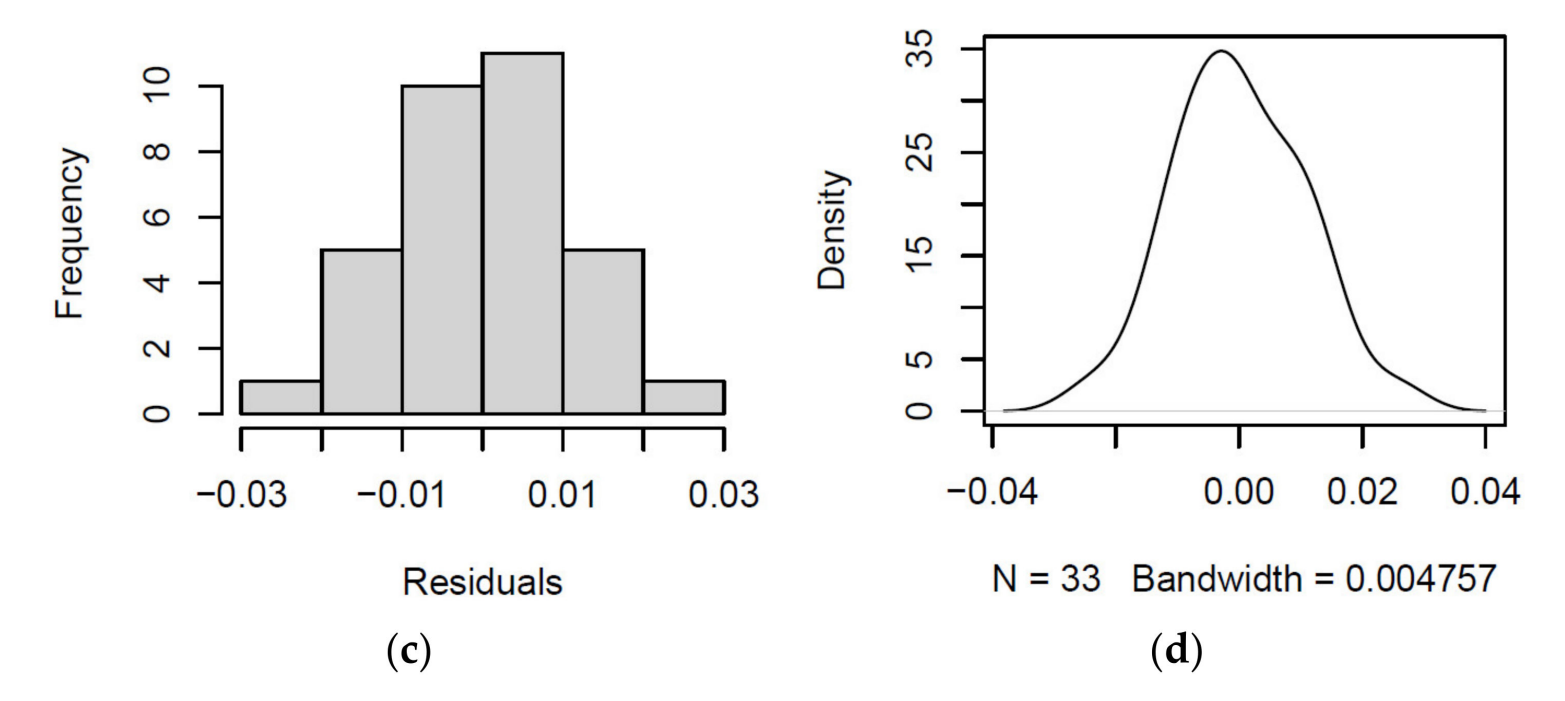
Residue analysis: (**a**) Normal Q–Q Plot; (**b**) Box Plot; (**c**) Histogram; (**d**) Density.

Due to the use of pseudo-central points, we performed a model verification near the central level of factor *x*_3_. Verification was performed by analysing the residues of the model for measurements with the stamping size *Z* = 316.08 mm. A graphical representation of the residue analysis is shown in Figure 14 and a summary in Table 13.

It follows from the above that the residues have a normal distribution, which was confirmed by the Shapiro–Wilk test (*p* = 0.9979) and the Kolmogorov–Smirnov test (*p* = 0.9406). We, therefore, verified the correctness of the model in the vicinity of the central level of factor *x*_3_.

**Figure 15:**
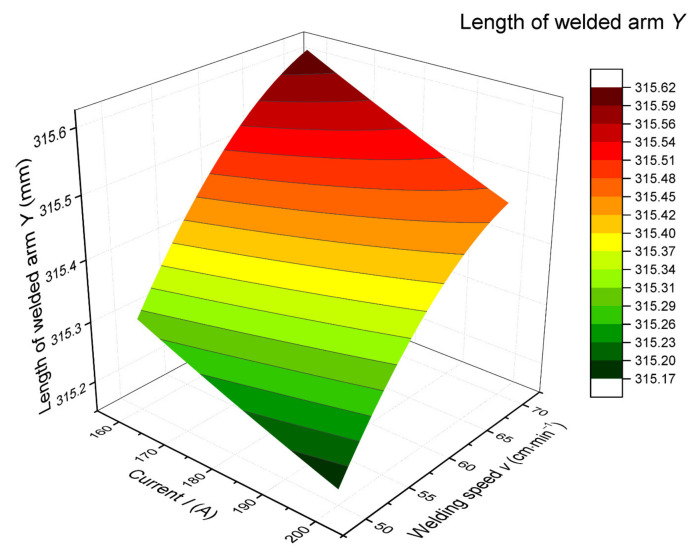
Dependence of the welded arm length on the current and the welding speed for the dimension of the stamping *Z* = 315.80 mm.

The output of the model, the graphic dependence of the length of the welded arm on the change of the current, and the welding speed for the dimension of the stamping *Z* = 315.80 mm are shown in Figure 15. 

**Figure 16:**
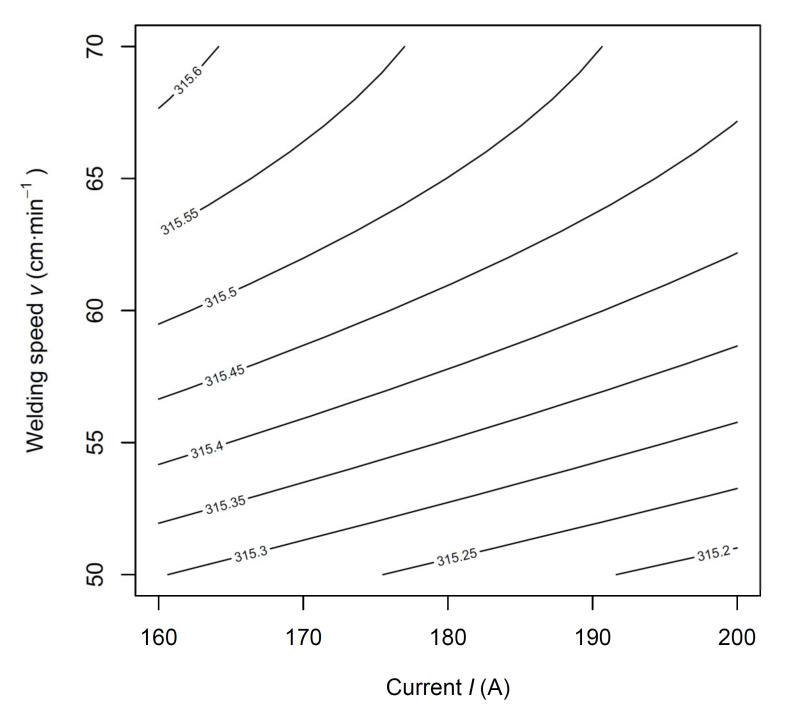
The dependence of the welded arm resulting length on the current and the welding speed for the dimension of the stamping Z = 315.80 mm.

The model confirms the theoretical assumptions, i.e., that minimal deformations (larger dimension of the welded arm) can be achieved at lower currents and higher welding speeds. The choice of optimal welding parameters can be processed in the form of recommendations for individual dimensions of stampings within the examined interval into a tabular form or graphically in the form of prediction maps. An example for the same dimension of the stamping is shown in Figure 16.

## 4. Conclusions

This article deals with a significant problem typical of the automotive industry—the dimensional accuracy of welded stampings. We have used the example of a specific component to introduce a new procedure, the result of which is a model that allows the use of deformation during welding to eliminate inaccuracies arising during the stamping process partially. The results can be summarized as follows:

- Methodology that has not been used in this area so far, i.e., the use of pseudo-central points and face-centred axial points in CCD under the assumption of a linear influence of the factor *x*_3_ on the response in the subsequent statistical analysis and modelling allowed us to create a model with a high value of the adjusted coefficient of determination.
- The use of the proposed model allowed us to increase the accuracy of the production or maximise the production while maintaining the required dimensions.
- The influence of basic process parameters, namely welding current and welding speed combined with the changing size of the stamping, was verified.
- The observed linear dependence of thermal deformation on the welding current in combination with a significant curvature of the effect of welding speed (Figure 13) illustrated the complexity of the problem from a physical point of view, as the heat introduced was energy of dissipative nature, which depended not only on the magnitude of the current flowing through and on the time for which the heating process takes place but also on the resistance that the conductive material puts.
- As the indirect measurement of the generated heat for the purpose of precise continuous control is very difficult even with the use of precise measuring instruments, simplified models based on heat input (e.g., *I*/*v*) should be implemented only where there are no high demands on model accuracy.
- The unambiguous result of the presented article confirms the theoretical, qualitative assumption that the selected operating parameters have a direct and measurably significant effect on the resulting deformation of the part.
- A specific benefit of the article is the coded Equation (2) with a description and graphical representation of the influence of individual factors (Figure 13) on the final dimension of the welded part.
- The new Equation (3) allows predicting the resulting dimension directly from the entered operating parameters within the considered interval. It is possible to use prediction maps (Figure 16) to set the monitored process optimally in practice.

## Figures and Tables

**Table 1:** Chemical composition (weight %) of the tested material.

Tested Material	C (%)	Si (%)	Mn (%)	P (%)	S (%)
SGAFC590DP (2 mm thickness)	0.071	0.183	1.895	0.018	0.004

**Table 2:** Mechanical properties of the tested material.

Tested Material	Yield Strength (MPa)	Ultimate Tensile Strength (MPa)	Elongation (%)
SGAFC590DP (2 mm thickness)	405	643	28

**Table 3:** Welding parameters.

Parameter	Parameter Type	Marking	Unit
Current	Variable	*I*	A
Welding speed		*v*	cm·min^−1^
Voltage	Automatic	*U*	V
Gas/wire dosing		–	–
Technology	Constant	MIG	–
Shielding welding gas		Ar	–
Wire-diameter		*d* = 1.2	mm
Wire-type		KISWEL KC-25M	–
Location and order of welds		–	–
Clamping parts		–	–

**Table 4:** Levels of observed factors.

Coded Scale	Natural Scale	Factor Level		
		−1	0	1
*x*_1_	Current—*I* (A)	160	180	200
*x*_2_	Welding speed—*v* (cm·min^−1^)	50	60	70
*x*_3_	Stamping size—*Z* (mm)	315.78	–	316.22

**Table 5:** Standard levels of observed factors.

Factor	*I* (A)	*v* (cm·min^−1^)	*Z* (mm)
Level	200	70	Variable over series

**Table 6:** Experiment levels and verification levels.

Factor	Levels Involved in the Model	Levels Not Involved in the Model (Model Verification)
*x*_1_	160, 180, 200	–
*x*_2_	50, 60, 70	52, 54, 56, 58, 62, 64, 66, 68 for all levels of factors *x*_1_ and *x*_3_
*x*_3_	315.78, 316.22	316.08

**Table 7:** Results of Kruskal–Wallis analysis of variance of individual parameters of the experiment.

Current—*I* (A)	Valid N	Sum of Ranks	Mean Rank
160	33	1950.50	59.1061
180	33	1645.00	49.8485
200	33	1354.50	41.0455
**Welding speed—*v* (cm∙min^−1^)**	**Valid N**	**Sum of Ranks**	**Mean Rank**
50	9	246.00	27.3333
52	9	286.50	31.8333
54	9	336.00	37.3333
56	9	393.50	43.7222
58	9	435.00	48.3333
60	9	476.00	52.8889
62	9	510.50	56.7222
64	9	530.50	58.9444
66	9	553.00	61.4444
68	9	579.00	64.3333
70	9	604.00	67.1111
**Stamping size—*Z* (mm)**	**Valid N**	**Sum of Ranks**	**Mean Rank**
315.78	33	596.00	18.0606
316.08	33	1829.00	55.4242
316.22	33	2525.00	76.5152

**Table 8:** Statistically significant differences in the arm length at different levels of factor *x*_2_.

Deformation of the Welded Arm (mm)		Increased Factor Level *x*_2_			
		64	66	68	70
The original level of factor *x*_2_	50	0.24	0.26	0.28	0.30
	52	–	0.22	0.24	0.26

**Table 9:** Suitability of the used model.

Term	Value
RSquare	0.974
RSquare Adj	0.969
Root Mean Square Error	0.044
Mean of Response	315.612
Observations (or Sum Wgts)	26.000

**Table 10:** ANOVA table of the model applied.

Source	DF	Sum of Squares	Mean Square	F Ratio	Prob > F
Model	4	1.565	0.391	197.661	<0.0001
Error	21	0.041	0.002	–	–
C. Total	25	1.606	–	–	–

**Table 11:** Insufficient model fit error testing.

Source	DF	Sum of Squares	Mean Square	F Ratio	Prob > F	Max RSq
Lack of Fit	13	0.002	<0.001	0.037	1.000	0.976
Pure Error	8	0.039	0.005	–	–	–
Total Error	21	0.041	–	–	–	–

**Table 12:** Estimation of model parameters.

Term	Estimate	Std Error	t Ratio	Prob > |t|	Lower 95%	Upper 95%
Intercept	315.634	0.012	26548.000	<0.0001 *	315.609	315.659
*x*_1_	−0.069	0.013	−5.390	<0.0001 *	−0.096	−0.042
*x*_2_	0.151	0.013	11.740	<0.0001 *	0.124	0.177
*x*_2_·*x*_2_	−0.048	0.018	−2.710	0.013 *	−0.084	−0.011
*x*_3_	0.217	0.009	24.830	<0.0001 *	0.198	0.235

* Significant at the level of significance 5%.

**Table 13:** Summary of residuals.

Term	Value
Minimum	−0.024
1st quartile	−0.008
Median	0
Mean	0
3rd quartile	0.008
Maximum	0.026
RSquare	0.991
RSquare Adj	0.991

## Data Availability

The data presented in this study are available on request from the corresponding author after obtaining permission of authorized person.
